# Development of Antibody‐Directed Therapies: *Quo Vadis*?

**DOI:** 10.1002/anie.201712185

**Published:** 2018-01-17

**Authors:** Tiago Rodrigues, Gonçalo J. L. Bernardes

**Affiliations:** ^1^ Instituto de Medicina Molecular Faculdade de Medicina da Universidade de Lisboa Av Prof Egas Moniz 1649-028 Lisboa Portugal; ^2^ Department of Chemistry University of Cambridge Lensfield Road CB2 1EW Cambridge UK

**Keywords:** anti-tumor agents, antibody–drug conjugates, bioconjugation, drug delivery, linkers

## Abstract

**Less is more**: The efficacy of antibody–drug conjugates (ADCs) for cancer therapy is traditionally associated with cleavable linkers for payload release. Evidence now suggests that simpler constructs without cleavable moieties can afford more stable and effective ADCs.

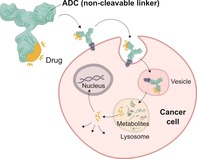

Cancer is a leading cause of mortality worldwide, and life‐changing drugs and therapeutic regimens are urgently needed. While natural products and synthetic molecules have contributed to the management of different cancers, it is known that their inherent cytotoxicity yields narrow therapeutic windows. Hence, several potentially useful drugs have been discontinued from development pipelines or seen limited clinical application.^1^

Targeted therapies afford viable solutions to finally fulfill Paul Ehrlich's century‐old “magic bullet” promise. Newly developed materials, nanotechnologies, and antibody–drug conjugates (ADCs) have the potential to reshape the treatment of aggressive and otherwise poorly manageable cancers. Indeed, ADCs couple the specific recognition of tumor‐associated antigens by antibodies to the high cytotoxicity of payloads to afford an active targeting construct. To date, four ADCs have been approved for clinical use—Adcetris, Kadcyla, Besponsa, and Mylotarg—and more than 60 are undergoing clinical trials.^2^ Despite this being a mature concept, much can be improved in future ADCs. For example, tuned linker technologies and advanced site‐specific conjugation chemistry can strongly influence the drug–antibody ratio, solubility, pharmacokinetics, and ultimately ADC efficacy. To that end, efforts have been made for the discovery of reactions leading to stable and homogeneous ADCs.^3^

The development of cleavable linkers, and the engineering of releasing mechanisms for them, has been considered essential for appropriate bioactivity of the payload in a disease setting. While acid‐labile hydrazone linkers have historical importance, more recently, two releasing mechanisms have been exploited, taking into account the fact that antibodies are internalized once bound to the antigen followed by lysosomal degradation: 1) a disulfide linkage is reduced in the presence of biological thiols such as glutathione with subsequent release of the payload and 2) a valine‐citrulline linker is cleaved by a protease (e.g. cathepsin B) to release the payload (Figure 1). These conditionally stable moieties were designed for intracellular delivery of the unmodified payload, and selectively kill the cancer cell and its diseased neighbors through the so‐called bystander effect, without harming healthy tissues. The natural product realm has been prolific in providing viable payloads for ADC research. Still, their modes of action remain limited. Typically, payloads are peptidic and/or macrocyclic and act through interference with either tubulin or DNA. However, emerging payload classes now include camptothecin and pyrrolobenzodiazepines. It is now also known that non‐internalizing ADCs can afford equally effective constructs for cancer therapy.^4^ Indeed the tumor microenvironment is also rich in payload‐releasing triggers that ought to be explored in depth. Similarly, ADCs may be best employed for liquid tumors where permeation of a rather bulky construct is not a limiting factor.

**Figure 1:**
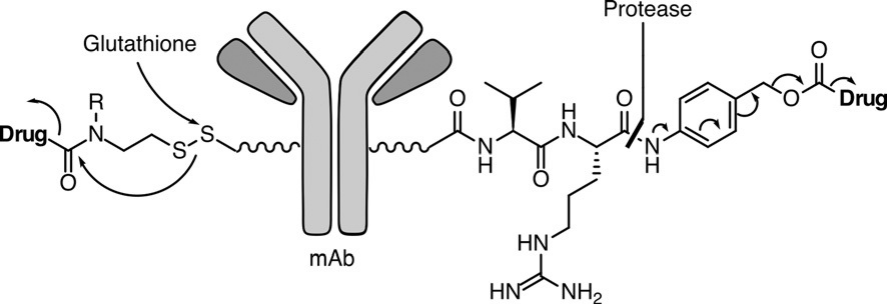
Common linkers used in ADCs and their drug‐release mechanisms.

Despite the payload release‐by‐design nature of ADCs, undesired drug bleaching has been commonly observed, resulting in untargeted drug delivery and toxicity. A leading cause for such premature payload liberation stems from the maleimide conjugation chemistry, since maleimides are prone to retro‐Michael additions.^5^ Consequently, improving overall ADC stability remains a topic of intense research.

Recently,^6^ researchers at Genentech have shown that suppression of the protease cathepsin B through CRISPR‐Cas9 gene deletion or shRNA knockdown had no statistically significant effect on the anticancer activity of monomethyl auristatin E (MMAE) in ADCs featuring the cleavable valine‐citrulline linker (*S*‐configured citrulline). The observation was reproducible in different cell lines with different degrees of intracellular accumulation of the tool constructs. In fact, mass spectrometry analyses suggested that other cysteine cathepsins are able to cleave the abovementioned linker with differing levels of efficiency.^6^ This observation further complements the recent finding that the valine‐citrulline linker is cleaved by carboxyesterase 1c, a key player in extracellular cleavage of said linkers and promoter of reduced ADC efficacy.^7^ The result also supports functional redundancy within the cathepsin protease family and overlapping substrates that allow catalytic compensation whenever cathepsin B expression is either reduced or absent. Cathepsin S appears to be particularly efficient in cleaving the valine‐citrulline linker.^6^

Contrary to current thinking in ADC research, a fully stable construct featuring the non‐cleavable valine‐citrulline (*R*‐configured citrulline) linker counterpart resulted in significant anticancer activity (IC_50_ value of 0.063 μg mL^−1^ for the parental KPL‐4 cell line and 0.085 μg mL^−1^ for cells not expressing cathepsin B). Although the valine‐(*R*)‐citrulline–MMAE ADCs are only around 50 % as potent as the *S*‐configured controls, their anticancer activity is still much higher than anticipated.^6^ What are then the underlying mechanisms of MMAE release and action? The data clearly show that lysosomal catabolism is robust in the sense that it liberates bioactive metabolites, through multiple mechanisms, from ADCs. Of note, the cysteine adduct of valine‐(*R*)‐citrulline–MMAE could be identified as the major catabolite from an ADC with a non‐cleavable linker.^6^ One must wonder whether the strategy can be generally applicable? We reason that ADCs with non‐cleavable linkers may be useful for internalizing antibodies. One can also envisage the extrapolation of such constructs to afford antibody–antibiotic conjugates. Otherwise there is no guarantee that the resulting metabolites are the same as those originating inside the lysosome and questions remain about their membrane permeability (Figure 2).

**Figure 2:**
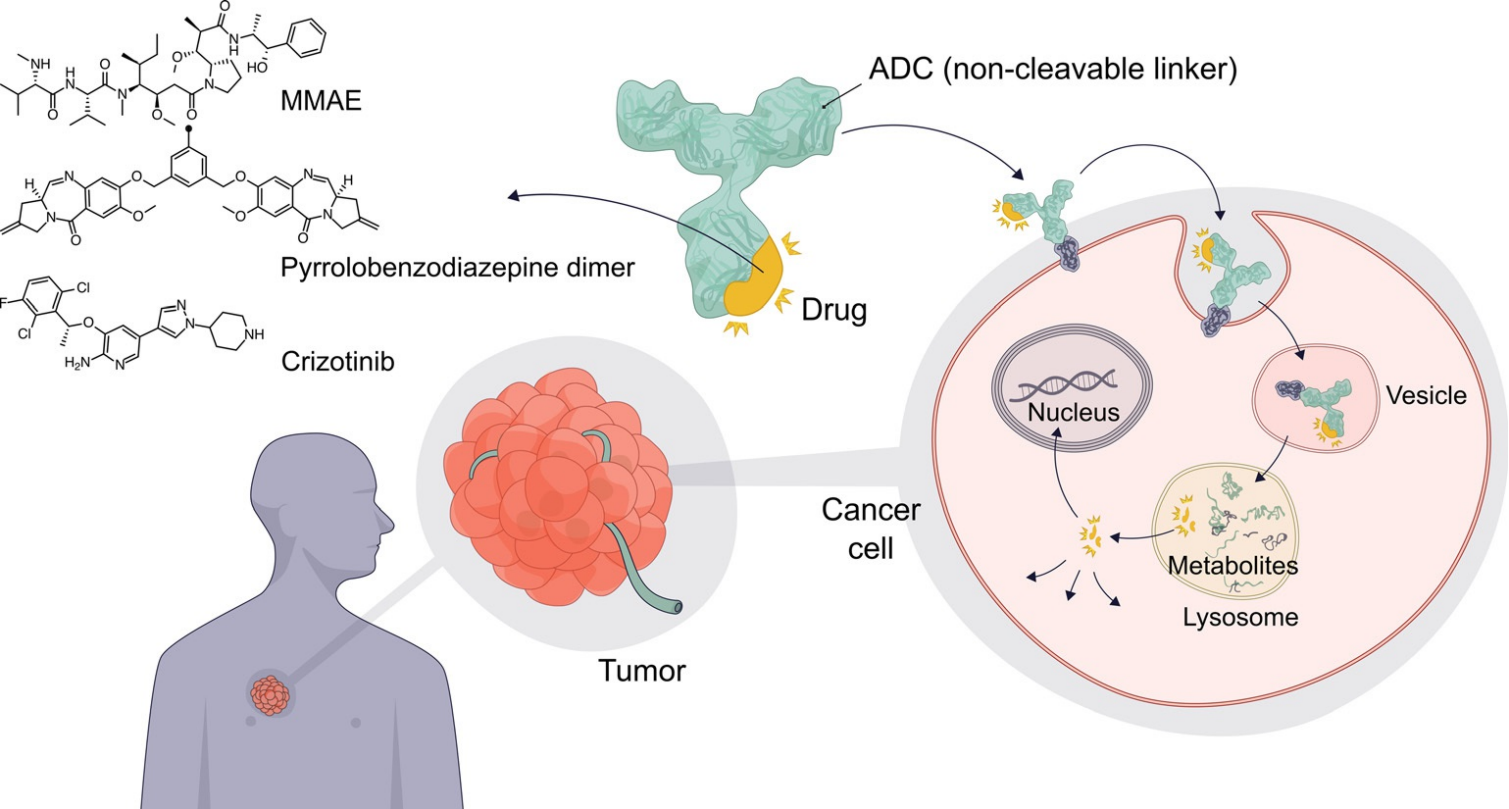
ADCs with non‐cleavable linker and release mechanism of the payloads. Payloads for which the strategy has been validated are depicted. Image compiled by Claudia Flandoli.

Genentech has also built anti‐HER2 ADCs carrying pyrrolobenzodiazepine dimers attached via non‐cleavable alkyne, triazole, and piperazine moieties.^8^ Iterative computer‐assisted drug design and synthesis efforts resulted in entities that likely preserved the intended binding mode of the dimer to the DNA minor groove. Subsequently, antibodies were engineered with two cysteine residues to afford a drug/antibody ratio of 2, the homogeneity of which was confirmed by LC–MS. The constructs displayed varying potencies across the model cell lines, with values in the low ng mL^−1^ range. Most importantly, the constructs showed dose‐responsive efficacy (0.3–6 mg kg^−1^) in the HER‐positive Founder 5 mammary tumor transplant mouse model, which correlated with the in vitro assay data. Overall, the results support the validity of such ADCs as cancer therapeutics. Moreover, they raise question marks over which variables are indeed relevant for optimization for the purpose of improving the cell‐killing activity of ADCs. While linkers play an important role in modulating the physicochemical/pharmacokinetic properties of the constructs, it is now clearer than ever that cellular bioactivity is more connected to the employed antibodies and payloads.

Linking drugs directly to antibodies holds great promise and can be applicable to ligands of diverse target families. For example, crizotinib, a kinase inhibitor, was directly conjugated through aza‐Michael ligation to dehydroalanine. The construct showed a 10‐fold activity improvement when compared to the small molecule against SKBR3 breast cancer cells.^9^

It is however important to retain a healthy skepticism. For example, payloads should be sufficiently potent to ensure drug‐target saturation and avoid undesirable antibody loading that could lead to aggregation. Also, what is the magnitude of the bystander effect for linkerless ADCs? With some pharmaceutical companies opting out of the ADC field, it is clear that numerous challenges and drawbacks might be expected. Nonetheless, we foresee that linkerless ADCs will be explored more frequently in the future to exploit payloads with differentiated modes of action. Taking into account the recent track record, the resulting constructs could provide therapeutic alternatives that are at least as effective as the cleavable counterparts but with minimal manipulation and improved stability.

## Conflict of interest

The authors declare no conflict of interest.
